# Glycyrrhizin Inhibits SARS-CoV-2 Entry into Cells by Targeting ACE2

**DOI:** 10.3390/life12111706

**Published:** 2022-10-26

**Authors:** Ming-Feng He, Jian-Hui Liang, Yan-Ni Shen, Jin-Wen Zhang, Ying Liu, Kuang-Yang Yang, Li-Chu Liu, Junyi Wang, Qian Xie, Chun Hu, Xun Song, Yan Wang

**Affiliations:** 1 Foshan Hospital of Traditional Chinese Medicine, Foshan 528000, China; 2 Center for Translation Medicine Research and Development, Shenzhen Institutes of Advanced Technology, Chinese Academy of Sciences, Shenzhen 518055, China; 3 Key Laboratory of Structure-Based Drug Design & Discovery, Ministry of Education, School of Pharmaceutical Engineering, Shenyang Pharmaceutical University, Shenyang 110016, China; 4 College of Pharmacy, Shenzhen Technology University, Shenzhen 518118, China; 5 Bluewood Associates Co., Ltd., Suzhou 215134, China

**Keywords:** SARS-CoV-2, ACE2, glycyrrhizin, spike protein RBD

## Abstract

Coronavirus Disease 2019 (COVID-19) is a highly infectious and pathogenic disease caused by the severe acute respiratory syndrome coronavirus 2 (SARS-CoV-2). Early in this epidemic, the herbal formulas used in traditional Chinese medicine (TCM) were widely used for the treatment of COVID-19 in China. According to Venn diagram analysis, we found that *Glycyrrhizae Radix et Rhizoma* is a frequent herb in TCM formulas against COVID-19. The extract of *Glycyrrhizae Radix et Rhizoma* exhibits an anti-SARS-CoV-2 replication activity in vitro, but its pharmacological mechanism remains unclear. We here demonstrate that glycyrrhizin, the main active ingredient of *Glycyrrhizae Radix et Rhizoma*, prevents the coronavirus from entering cells by targeting angiotensin-converting enzyme 2 (ACE2). Glycyrrhizin inhibited the binding of the spike protein of the SARS-CoV-2 to ACE2 in our Western blot-based assay. The following bulk RNA-seq analysis showed that glycyrrhizin down-regulated ACE2 expression in vitro which was further confirmed by Western blot and quantitative PCR. Together, we believe that glycyrrhizin inhibits SARS-CoV-2 entry into cells by targeting ACE2.

## 1. Introduction

The severe acute respiratory syndrome coronavirus 2 (SARS-CoV-2) is a positive-sense, single-stranded RNA virus belonging to the Betacoronavirus B lineage and is closely related to the SARS-CoV [1]. Viral pneumonia caused by the SARS-CoV-2 was named as coronavirus disease 2019 (COVID-19) by the World Health Organization in February 2020. COVID-19 is highly contagious and has spread rapidly around the world [2,3]. Severely ill patients may develop dyspnea and hypoxemia, and even develop acute respiratory distress syndrome, etc. [4,5].

Currently ongoing clinical drug trials for the treatment of COVID-19 patients mainly include: (1) viral protease inhibitors; (2) viral entry inhibitors; (3) RNA-dependent RNA polymerase inhibitors; (4) immune modulators; (5) monoclonal antibodies; (6) Janus kinase inhibitors [6,7,8]. In addition to synthetic drugs, the effectiveness of traditional Chinese medicine (TCM) formulas in combating COVID-19 has been confirmed by clinical data [9]. TCM formulas and Chinese patent drugs (CPDs) listed in the Chinese Clinical Guidance for COVID-19 Pneumonia Diagnosis and Treatment have been reported to benefit COVID-19 patients in China [10].

TCM has a long history and rich experience in preventing infectious diseases. It has definite efficacy in improving clinical symptoms, shortening recovery time and reducing the rate of severe diseases [11]. A TCM formula may contain hundreds of phytochemicals and target hundreds of proteins in multiple host cells. Therefore, the identification of active ingredients from TCM by using traditional bioactive-tracing methods has become a time-consuming endeavor. Molecular docking can accurately predict the binding energy and mode between small molecules and the target protein, providing a new approach for drug screening [12]. Recently, some phytochemicals from TCM formulas have been reported to exhibit anti-SARS-CoV-2 activities in vitro. However, how these phytochemicals inhibit virus replication is not yet unveiled.

In this study, we complied 714 phytochemicals from twelve TCM formulas from the Diagnosis and Treatment Scheme of Novel Coronavirus for molecular docking screening. Glycyrrhizin, the predominant active ingredient from *Glycyrrhizae Radix et Rhizoma,* exhibited high binding affinity with angiotensin-converting enzyme 2 (ACE2) and main protease (M^pro^) in our docking screening. Since we identified *Glycyrrhizae Radix et Rhizoma* as a frequent herb in TCM formulas against the SARS-CoV-2, we speculated that glycyrrhizin inhibits coronavirus replication by directly targeting ACE2 and M^pro^. The following biochemical assay showed that glycyrrhizin was not able to inhibit M^pro^ activity but strongly inhibit the interaction between the spike protein and ACE2. The further bulk RNA-seq analysis showed that glycyrrhizin down-regulated ACE2 expression in vitro which was evidenced by Western blot and quantitative PCR. Together, we believe that glycyrrhizin inhibits coronavirus replication by targeting ACE2.

## 2. Materials and Methods

### 2.1. Ligand Library Preparation

All chemical components of some traditional Chinese medicine formulas in the Diagnosis and Treatment Scheme of Novel Coronavirus were identified using the Traditional Chinese Medicine Systems Pharmacology Database and Analysis Platform (TCMSP, https://tcmsp-e.com/ (accessed on 6 June 2020)). The herbs contained in five classical Chinese medicine prescriptions against SARS-CoV-2 were drawn into a Venn diagram.

The SMILE formula of each benchmark ligand and small molecular compound was obtained from PubChem (http://pubchem.ncbi.nlm.nih.gov/ (accessed on 1 February 2021)). Using Corina website (https://www.mn-am.Com/online_demos/corina_demo (accessed on1 February 2021 )), we converted SMILE into the PDB format. Finally, the downloaded PDB files of ligands were converted into the PDBQT format by AutoDockTools-1.5.6 (The Scripps Research Institute, La Jolla, CA, USA).

### 2.2. Protein Model Preparation

The PDB format of the target protein model was obtained from Protein Data Bank (https://www.rcsb.org/ (accessed on 1 February 2021)) and then opened in AutoDockTools-1.5.6 (The Scripps Research Institute, La Jolla, CA, USA). Grid box of AutoDockTools was used to include the binding sites of the target protein and the potential ligand, which was later removed from the three-dimensional structure of the protein model. With the editing function of AutoDockTools, the PDB file was output after removing water molecules from the protein structure and adding hydrogen bonds.

### 2.3. Component–Target Molecular Docking

We first downloaded the 3D structure of key target proteins from the PDB database (http://www.rcsb.org/ (accessed on 1 February 2021)), then used the AutoDock Tools (version 1.5.6) for protein isolation and modification. The docking analysis of prepared ligands and target proteins was performed byAutodock vina (The Scripps Research Institute, La Jolla, CA, USA). By using grid box, ligands in the molecular library were placed into previously identified binding sites of the target protein and predicted the docking binding energy. If the docking binding energy is less than 0 kcal/mol, the ligand and the receptor can spontaneously bind, whereas if the binding energy is less than −7 kcal/mol, it indicates that the binding force is strong [13].

### 2.4. M^pro^ Enzyme Activity Inhibition Test

We used a commercial kit (2019-nCoV M^pro^/3CL^pro^ inhibitor screening kit, P0312S, Beyotime Biotech, Shanghai, China) based on fluorescence resonance energy transfer (FRET) to identify whether the screened compounds have an inhibitory effect on the activity of SARS-CoV-2 M^pro^. Edans is a fluorescence donor and Dabcyl is a fluorescence receptor. Edans and Dabcyl are connected to both ends of the natural substrate of SARS-CoV-2 M^pro^, and when the natural substrate is cut by enzyme, Edans fluorescence can be detected. The maximum excitation wavelength of Edans is 340 nm and the maximum emission wavelength is 490 nm. We determined the 50% inhibitory concentration (IC_50_) by plotting the relative fluorescence units versus the compound concentration. Results are reported as IC_50_ values. Ebselen worked as a positive control.

### 2.5. Biolayer Interferometry (BLI) Assay

The binding affinity of glycyrrhizin and His-tagged SARS-CoV-2 S protein was measured by Ni-NTA biosensors (Forte Bio, 180029). Both 1 μM of SARS-CoV-2 S protein and glycyrrhizin (final concentration at 125 μg/mL, 250 μg/mL and 500 μg/mL) were diluted with the running buffer (PBS, 0.02% Tween-20, and 0.1% BSA) [14]. The sensor was pre-wetted in the buffer for 10 min, and then the baseline was equilibrated for 30 s. Next, the S protein of SARS-CoV-2 was loaded to saturation for 4 min following by washing the sensor with running buffer for 2 min. We then immersed the sensor in glycyrrhizin solution for 4 min for binding followed by dissociation in PBS for another 4 min. The K_d_ value was calculated by using a 1:1 binding model and the Octet System Data Analysis Software Version 7 (ForteBio, Inc., Menlo Park, CA, USA).

### 2.6. Cell Culture

Vero E6 cells (Guangzhou Huatuo Biological Technology Co. Ltd., Guangzhou, China) were cultured in Dulbecco’s modified Eagle’s medium (DMEM, Corning, NY, USA) containing 10% fetal bovine serum, 1% penicillin/streptomycin and 0.1% amphotericin B at 37 °C and 5% CO_2_. When the cells grew to the proper confluence in the culture dish, the cells were digested and counted, and then the cells were seeded on a 6-well plate for the experiment.

### 2.7. RNA Sequencing

In order to investigate the regulation of glycyrrhizin on genes and pathways related to SARS-CoV-2 in Vero E6 cells, the cells were treated according to the experimental protocol, and the total RNA of the cells was extracted and analyzed by gene sequencing. Org.Hs.eg.db (Version 3.8.2, https://bioconductor.org/packages/release/data/annotation/html/org.Hs.eg.db.html, accessed on 1 February 2021 ) and ClusterProfiler (Version 3.9, http://dk.archive.ubuntu.com/bioconductor/packages/3.9/bioc/html/clusterProfiler.html, accessed on 1 February 2021 ) packages in R (Version 3.6.2, https://www.r-project.org/, accessed on 1 June 2018) were used to analyze Kyoto Encyclopedia of Genes and Genomes (KEGG) pathway.

### 2.8. Western Blot Analysis

The protein samples were extracted with RIPA buffer (Thermo scientific, 89900, Waltham, MA, USA). After centrifuging the samples, part of the supernatant was taken out to determine the concentration of the samples by using BCA kit (Beyotime, P0012, Shanghai, China). After that, we mixed the remaining supernatant and protein-loading buffer (Meilunbio, MA0003-D, Dalian, China) in a 4:1 volume ratio, then boiled them at 95 °C for 5 min. Samples were separated by 10% sodium dodecyl sulfate–polyacrylamide gel electrophoresis gel (Beyotime, P0012A, Shanghai, China), and then the proteins were transferred to a PVDF membrane activated by methanol. The membrane was then incubated with primary antibody at 4 °C overnight. After washing the membrane with 1× TBST solution, the blots were incubated with secondary antibodies for one hour at room temperature. After washing the membrane three times with 1× TBST solution, we used an ECL reagent kit (Solarbio, PE0010, Beijing, China) to visualize the target proteins on the membrane through chemiluminescence.

### 2.9. RNA Preparation and Real-Time Quantitative Polymerase Chain Reaction (RT-qPCR)

After washing cells with cold PBS solution, RNAiso Plus reagent (Takara Bio, Kusatsu, Japan) was added to extract total RNA in cells. The NanoDrop 2000 (Thermo Scientific, Waltham, MA, USA) was used to measure the concentration and purity of RNA. Total RNA (2 μg) was reverse-transcribed with the PrimeScript RT Master Mix (Takara Bio; Kusatsu, Japan). Levels of mRNA were evaluated by qPCR amplification on a LightCycler96 real-time fluorescence qPCR instrument (Roche, Basel, Switzerland). The RT-qPCR amplification system includes forward/reverse primers (0.5 μL each), 3 μL of sterile deionized distilled water, 5 μL of SYBR Green Premix (Accubate Biology, Changsha, Hunan Province, China) and cDNA templates (1 μL). GAPDH was used as an internal control. The primer sequence of the target gene is as follows: GAPDH: forward: 5′-ATGACCCCTTCATTGACC-3′, reverse: 5′-GAAGATGGTGATGGGATTTC-3′; ACE2: forward: 5′-ACAGTCCACACTTGCCCAAAT-3′, 5′-TGAGAGCACTGAAGACCCATT-3′. All reactions were performed in triplicate. The relative expression of target genes was calculated using the 2^−ΔΔCt^ method.

## 3. Results and Discussion

### 3.1. Glycyrrhizae Radix et Rhizoma Is a Frequent Herb in TCM Formulas against SARS-CoV-2

**Figure 1:**
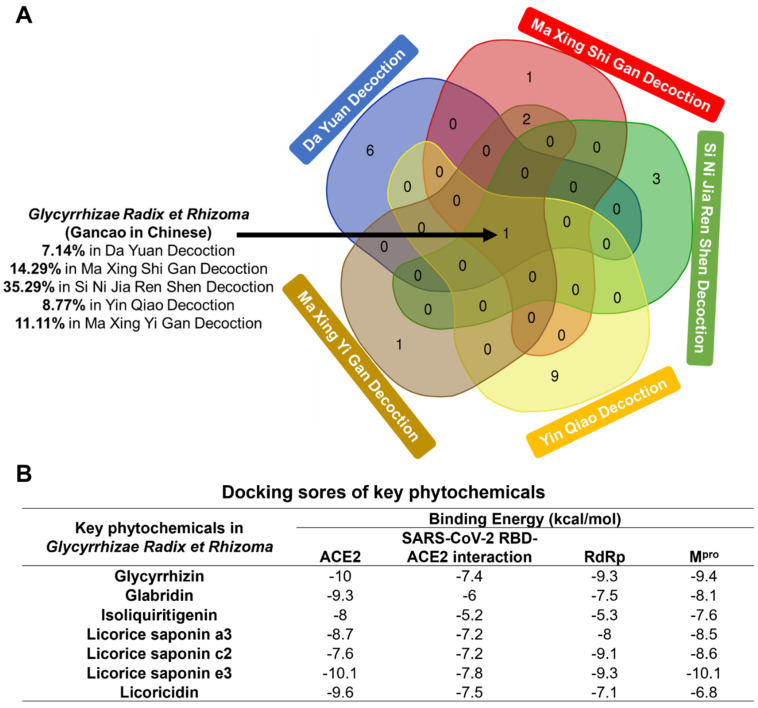
*Glycyrrhizae Radix et Rhizoma*, as the main component, exists in five traditional Chinese medicine prescriptions against novel coronavirus at the same time. (**A**) Venn diagram of herbs contained in five anti-SARS-CoV-2 Chinese herbal formulas. The numbers in intersecting part represent the numbers of shared herbs within decoctions. Gancao exists in five traditional Chinese medicine formulations at the same time, the contents of which are listed separately. (**B**) Docking scores of key phytochemicals in *Glycyrrhizae Radix et Rhizoma* with four potential targets of SARS-CoV-2.

The major phytochemicals in the traditional Chinese formulas against the SARS-CoV-2 are summarized in Table S1. The herbs of the five traditional Chinese formulas were drawn into a Venn diagram (Figure 1A), showing that all of these formulas contain Glycyrrhizae Radix et Rhizoma. The content of Glycyrrhizae Radix et Rhizoma exceeds 10% in three formulas, indicating its key role against the SARS-CoV-2. Seven predominant phytochemicals in Glycyrrhizae Radix et Rhizoma were obtained from PubChem for following docking analysis. Four proteins related to the entry and replication of the SARS-CoV-2, including ACE2, RdRp and M^pro^, were selected as target proteins (Table S2). As shown in Figure 1B, the binding free energy of glycyrrhizin and licorice saponin e3 with ACE2 is −10 kcal/mol, indicating that these two active components can stably bind to the target molecule. These two compounds were also the highest-scoring phytochemicals in binding to RdRp. We screened these active compounds according to the criteria of oral bioavailability (OB) ≥ 30%, drug-likeness (DL) ≥ 0.18 [13] and the corresponding plasma concentration. Finally, glycyrrhizin was identified as the most potent ingredient in Glycyrrhizae Radix et Rhizoma against the coronavirus. Interestingly, glycyrrhizin has been reported to act as a weak inhibitor of the SARS-CoV-2 with EC_50_ at 530 μM [15]. However, how glycyrrhizin inhibits virus replication requires further exploration.

### 3.2. Glycyrrhizin Is Not a Strong M^pro^ Inhibitor of SARS-CoV-2

M^pro^ of the SARS-CoV-2 cleaves two polyproteins (pp1a/pp1ab) into individual nonstructural proteins that are required for viral genome replication [16,17,18].

**Figure 2:**
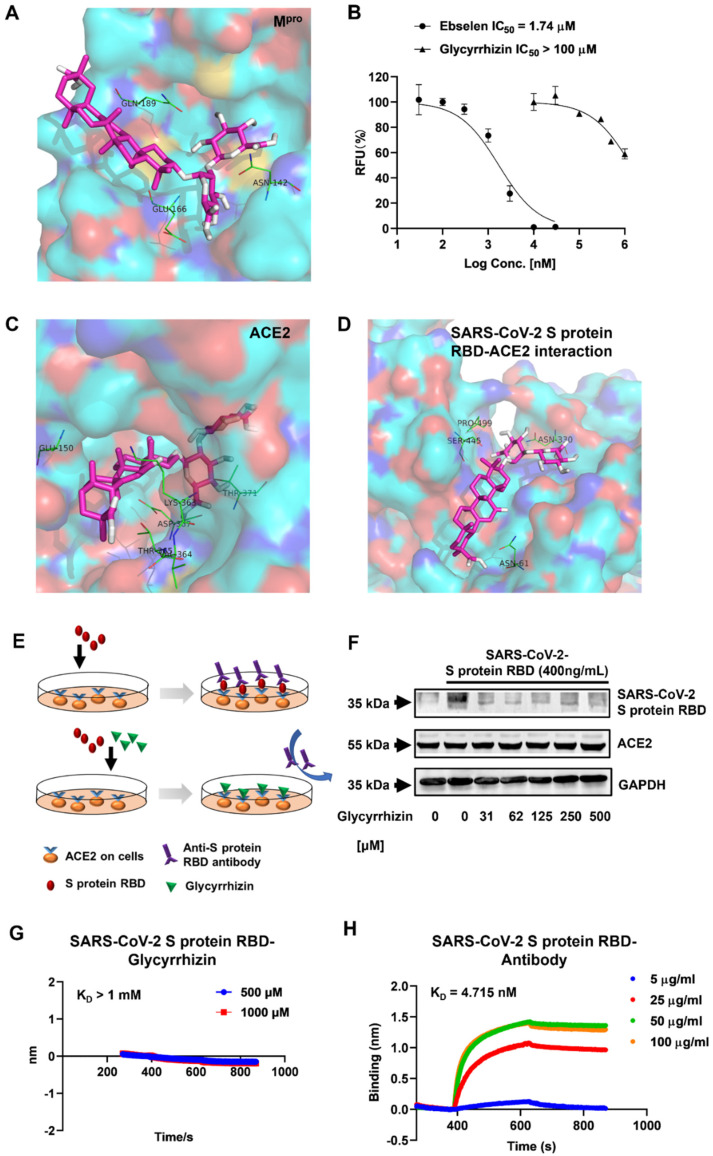
Glycyrrhizin is an effective substance against SARS-CoV-2. (**A**) Docking pose of glycyrrhizin (magenta sticks) bound to the catalytic site of SARS-CoV-2 M^pro^; the key M^pro^ interacting residues are represented by green, thin sticks. (**B**) Determination of glycyrrhizin inhibiting M^pro^ activity in biochemical assay, expressed by IC_50_ value. Ebselen as a positive control. (**C**) Docking poses and binding interactions of glycyrrhizin (magenta sticks) inside the human ACE2 active site. (**D**) The best conformation and binding interactions of the glycyrrhizin (magenta sticks) within ACE-2–RBD interface; the hot spot residues are represented by green, thin sticks and labeled in the inset. (**E**) Schematic diagram of glycyrrhizin preventing S protein RBD from binding to ACE2 receptor. When the SARS-CoV-2 attacks the cells, S protein RBD can bind to ACE2 receptor on the cell, and anti-S protein RBD antibody further binds to the protein. However, when glycyrrhizin and S protein RBD were added to the cultured Vero E6 cells at the same time, glycyrrhizin was more easily binded to ACE2, which made the antibody be added later without a protein that can specifically bind to it, and the antibody could not be detected in subsequent tests. (**F**) Vero E6 cells were treated with different concentrations of glycyrrhizin (one of which was 0) for 2 h, then the supernatant was discarded, and the cells were co-incubated for 2 h with the fresh medium containing 400 ng/mL of SARS-CoV-2 S protein RBD and different concentrations of glycyrrhizin. Following that, we extracted the proteins from these cell samples for Western blot analysis to determine the expression of ACE2 in Vero E6 cells and the content of cell-bound S protein. (**G**) The binding capacity of glycyrrhizin to SARS-CoV-2 S protein RBD. SARS-CoV-2 S protein RBD was immobilized on streptavidin sensors and reacted with the appropriate amounts of chrysin for biolayer interferometry (BLI). The apparent dissociation constants, K_D_ (M), were calculated and shown in the graph. Data are representative of two independent experiments. (**H**) Characteristics of the interaction between different concentrations of anti-S protein RBD antibody and S protein RBD. The test conditions are the same as figure **G**.

The inhibition of M^pro^ catalytic activity has been reported to inhibit coronavirus replication in cells [19]. According to our docking screening, glycyrrhizin exhibited a good binding affinity with M^pro^ (docking score −9.4 kcal/mol). Figure 2A shows that glycyrrhizin directly binds to Gln-189, Asn-142 and Glu-166 in M^pro^ catalytic pocket through hydrogen bonds and van der Waals force. In this way, the antiviral activity of glycyrrhizin may depend on its capability of inhibiting the enzymatic activity of M^pro^. Then, the anti-M^pro^ activity of glycyrrhizin was determined by an enzymatic assay based on FRET. The median inhibitory concentration (IC_50_) for the positive control Ebselen was calculated to be 1.74 μM. Glycyrrhizin, on the other hand, failed to achieve fifty percent inhibition until 1 mM (Figure 2B), indicating that the anti-SARS-CoV-2 activity of glycyrrhizin does not depend on its anti-M^pro^ activity.

### 3.3. Glycyrrhizin Inhibits SARS-CoV-2 Spike Protein–ACE2 Interaction

The S protein of the SARS-CoV-2 has been reported to attach to ACE2 on the cell membrane and then penetrate the host cells through ACE2 [20]. Therefore, the blockade of the S protein–ACE2 interaction has a good chance to inhibit coronavirus entry [21]. The inhibition of ACE2’s catalytic activity leads to the confirmation that ACE2 can inhibit S protein binding [22]. Additionally, compounds can directly bind to the ACE2–S protein binding site to inhibit virus entry [23].

In our docking simulation, glycyrrhizin directly binds to the catalytic pocket of ACE2 with high binding affinity (−10 kcal/mol) (Figure 1B). Glu-150, Thr-371 and Lys-361 are key residues for glycyrrhizin binding (Figure 2C). Furthermore, glycyrrhizin shows a mild binding affinity with the SARS-CoV-2 S protein/ACE2 binding interface (−7.4 kcal/mol, Figure 1B). Glycyrrhizin binds to Asn-61, Ser-445 and Asn-330 through hydrogen bonds (Figure 2D). We therefore speculated that glycyrrhizin could directly bind to ACE2 to inhibit SARS-CoV-2 entry.

We then designed a cell-based assay to test whether glycyrrhizin can inhibit the interaction of the SARS-CoV-2 S protein and ACE2. The Vero E6 cells were incubated at the receptor-binding domain (RBD) of the S protein. The S protein RBD can attach to Vero E6 cells through ACE2 binding. The attached S protein RBD on Vero E6 cell membranes can be probed by the RBD antibody and semi-quantified by Western blot. If glycyrrhizin inhibits the interaction of the SARS-CoV-2 S protein and ACE2, less S protein RBD on Vero E6 cell membranes will be observed in a Western blot assay (Figure 2E). The Western blot result shows that when the concentration of glycyrrhizin reached 31 μM, the content of S protein RBD in the cell lysate was significantly lower than that of the blank group (Figure 2F), raising the possibility that glycyrrhizin can inhibit the binding of the S protein RBD to the ACE2 receptor at this concentration. Furthermore, the expression of ACE2 did not show significant changes in the seven samples (Figure 2F).

We also tested whether glycyrrhizin directly binds to the S protein by using biolayer interferometry (BLI). The anti-S protein RBD antibody binds to the S protein RBD in a dose-dependent manner by BLI analysis (Figure 2G). The equilibrium dissociation constant (K_D_) between the S protein RBD and its antibody was calculated as 4.715 × 10^−9^ M, whereas there was little interaction between the S protein RBD and glycyrrhizin under comparable conditions (Figure 2H). These results indicate that glycyrrhizin has little chance of binding to the S protein RBD. Taken together, glycyrrhizin is likely to directly bind to ACE2 to prevent SARS-CoV-2 entry.

### 3.4. Glycyrrhizin Regulates Gene Expression in Host Cells

We found that glycyrrhizin can significantly inhibit the interaction of the S protein and ACE2 at 31 μM, but previous research showed that the inhibitory concentration of glycyrrhizin on the SARS-CoV-2 is greater than 500 μM [15]. Therefore, we speculated that glycyrrhizin may upregulate some genes in the host cells, which can promote SARS-CoV-2 entry or replication. To test our conjecture, bulk RNA sequencing was performed to identify critical genes regulated by glycyrrhizin.

**Figure 3:**
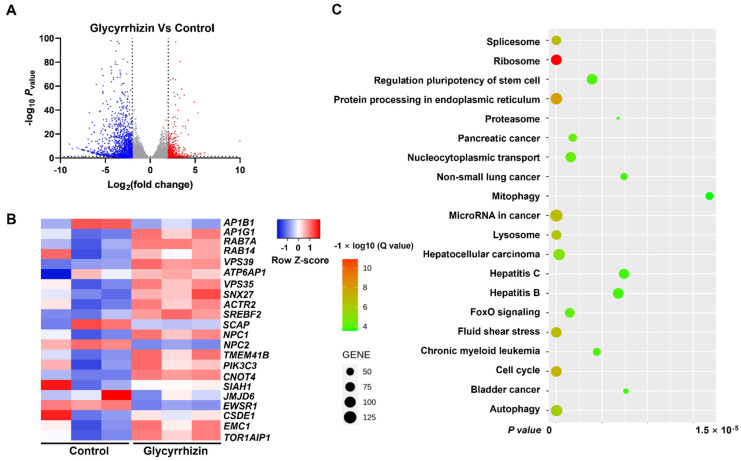
Glycyrrhizin regulates gene expression in Vero E6 cells. The Vero E6 cells were cultured in a six-well plate, and after the cells adhered and grew to 80–90% confluence, the supernatant was discarded and fresh medium with or without glycyrrhizin was added for 48 h of treatment. Then, the total mRNA of the cells was extracted for gene sequencing analysis. (**A**) Volcano plot displaying differentially expressed genes between glycyrrhizin and control groups (*n* = 3 biologically independent cell samples). Significant genes were called via Cuffdiff. The red dots represent the up-regulated expressed transcripts between glycyrrhizin and control; the blue dots represent the transcripts whose expression was down-regulated. (**B**) Heat map of differential expression of genes associated with SARS-CoV-2 infection in control and glycyrrhizin groups (*n* = 3 biologically independent cell samples; FDR < 0.001). Statistical tests were embedded in Cuffdiff. Each row represents a gene, red means increased gene expression, blue means decreased gene expression and the darker the color, the more obvious the trend is. (**C**) KEGG pathway enrichment analysis of key targets.

The volcano plot in Figure 3A shows the distribution of differential gene expressions between the two samples. The statistical results show that among the 17,768 genes detected, the expression of 643 genes in the treatment group was significantly up-regulated and 2004 genes were significantly down-regulated compared to the control group.

Next, we selected the genes that facilitate virus replication to make a heat map (Figure 3B), the horizontal axis of which represents different experimental samples, and the vertical axis of which represents different genes; blue represents a low gene expression level, and red represents a high gene expression level. For example, after treatment by glycyrrhizin, the expressions of *RAB7A, PIK3C3* and *EMC1* significantly increased. RAB7A performs virion assembly and release during viral replication; PIK3C3 supports DNA replication by inducing autophagy after HBx binding and EMC1 mediates ER-to-cytosol transport of virions during entry [24,25,26]. These up-regulated genes may facilitate SARS-CoV-2 replication. Together, the up-regulations of *RAB7A, PIK3C3* and *EMC1* by glycyrrhizin may reduce its antiviral activity in vitro.

In order to study the biological functions associated with these hypervariable genes, we did a KEGG pathway analysis. The enriched pathways were summarized in Figure 3C, the number of genes concentrated on the modified pathway is represented by the size of the dots and the significance of enrichment is represented by the color of these dots. KEGG pathway analysis showed that the ribosome, nucleocytoplasmic transport and hepatitis B pathway were significantly regulated by glycyrrhizin. However, these pathways have no obvious relationship with the replication of SARS-CoV-2 in cells, which was consistent with Gene Ontology (GO) enrichment analysis (Table S3). Interestingly, glycyrrhizin can regulate the forkhead box O (FoxO) signaling that is highly involved with interferon expression [27]. This indicates that glycyrrhizin may trigger the host’s immune system to fight the virus.

### 3.5. Glycyrrhizin Can Reduce the Expression of ACE2 In Vitro

**Figure 4:**
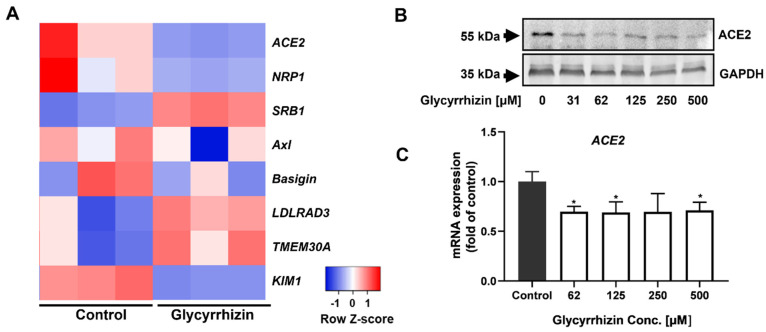
Glycyrrhizin can reduce the expression of ACE2 in cells. (**A**) Heat map of differentially expressed genes in groups control and glycyrrhizin, which are part of the genes required for SARS-CoV-2 entry (*n* = 3 biologically independent cell samples; FDR < 0.001). Statistical tests were embedded in Cuffdiff. (**B**) When the Vero E6 cells grow to 80–90% confluence in the well plate, the supernatant was discarded and cells were treated with the addition of fresh medium containing various concentrations or without glycyrrhizin for 48 h. After that, the total protein of the cells was extracted for Western blot analysis of the expression of ACE2 in the cells. (**C**) After treatment of Vero E6 cells with various concentrations of glycyrrhizin for 48 h, the cells were harvested and total RNA was extracted. After measuring the concentration of total RNA, reverse transcription and quantitative PCR were performed to measure mRNA expression levels of ACE2. Each independent experiment was repeated at least three times and presented as mean ± SD, *n* = 3. The results were analyzed through one-way ANOVA test (* indicates *p* < 0.05).

In addition to ACE2, other membrane receptors can help SARS-CoV-2 entry [28,29], and we also tested whether these entry receptors can be regulated by glycyrrhizin in a heat map analysis (Figure 4A). The results show that the expressions of *NRP1*, *Basigin* and *KIM1* were significantly down-regulated. The expressions of *SRB1*, *LDLRAD3* and *TMEM30A* were significantly up-regulated. Among the proteins encoded by these genes, NRP1 interacts with the SARS-CoV-2 S1 protein, which can also facilitate SARS-CoV-2 cell entry and infectivity [30]. Studies have demonstrated that the complex of kidney injury molecule-1 (KIM1) and RBD of the SARS-CoV-2 facilitates RBD attachment to the cell membrane [31]. Scavenger receptor class B type 1 (SRB1) also facilitates SARS-CoV-2 entry into ACE2-expressing cells by augmenting the adhesion of the virus [32]. Furthermore, the interaction of the SARS-CoV-2 spike protein with Low Density Lipoprotein Receptor Class A Domain Containing 3 (LDLRAD3) and Transmembrane Protein 30A (TMEM30A) can trigger membrane-to-membrane fusion [33].

Interestingly, we found that the expression of ACE2 was significantly decreased in the glycyrrhizin group. This indicates that glycyrrhizin can not only inhibit the binding of RBD to ACE2, but also reduce the expression of ACE2 in host cells. The following Western blot showed that glycyrrhizin significantly reduced the expression of ACE2 at a concentration of 31 μM (Figure 4B). RT-qPCR results (Figure 4C) show that when the concentration of glycyrrhizin reached 62 μM, its mRNA expression was also significantly down-regulated.

## 4. Conclusions

Through the Venn analysis of TCM against the SARS-CoV-2, we found that *Glycyrrhizae Radix et Rhizoma* is a frequently occurring herb. Glycyrrhizin, the predominate phytochemical in *Glycyrrhizae Radix et Rhizoma*, can prevent the SARS-CoV-2 from entering cells and replicating by reducing the expression of ACE2 and inhibiting the interaction between the S protein RBD and ACE2. However, through the results of bulk sequencing, we found that glycyrrhizin can up-regulate the expression of some other genes related to the promotion of virus entry into host cells, which might abolish the anti-SARS-CoV-2 activity of glycyrrhizin. However, the known serum concentration of glycyrrhizin is around 1 μM [34], which is much lower than its antiviral IC_50_ value. We estimate that in addition to directly inhibiting virus replication, glycyrrhizin may fight the SARS-CoV-2 through other indirect mechanisms. Together, we believe that glycyrrhizin inhibits SARS-CoV-2 entry into cells by targeting ACE2.

## Data Availability

The data presented in this study are available within the manuscript and in the Supplementary Materials. Further data can be provided upon request to the corresponding author.

## Supplementary Materials

The following supporting information can be downloaded at: https://www.mdpi.com/article/10.3390/life12111706/s1, Table S1: The molecule library of traditional Chinese formulas and Chinese patent drugs; Table S2: Target protein setup; Table S3: GO enrichment analysis.
