# Supplementary material for: Glycyrrhizin Inhibits SARS-CoV-2 Entry into Cells by Targeting ACE2
Source: Life (Basel). 2022 Oct 26;12(11):1706. doi: 10.3390/life12111706 (PMC9697465; doi:10.3390/life12111706)
Supplement: Supplementary file 1 [file life-12-01706-s001.zip › life-1930803-supplementary.pdf]

Article

Glycyrrhizin Inhibits SARS-CoV-2 Entry into Cells by Targeting ACE2

Ming-Feng He [1,†], Jian-Hui Liang [2,†], Yan-Ni Shen [2,3,†], Jin-Wen Zhang [2], Ying Liu [4], Kuang-Yang Yang [1], Li-Chu Liu [1], Junyi Danny Wang [5], Qian Xie [2], Chun Hu [3], Xun Song [4,*] and Yan Wang [2,*]

[1] Foshan Hospital of Traditional Chinese Medicine, Foshan 528000, China
[2] Center for Translation Medicine Research and Development, Shenzhen Institutes of Advanced Technology, Chinese Academy of Sciences, Shenzhen 518055, China
[3] Key Laboratory of Structure-based Drug Design & Discovery, Ministry of Education, School of Pharmaceutical Engineering, Shenyang Pharmaceutical University, Shenyang 110016, China
[4] College of Pharmacy, Shenzhen Technology University, Shenzhen 518118, China
[5] Bluewood Associates Co., Ltd., Suzhou 215134, China
* Correspondence: songxun@sztu.edu.cn (X.S.); yan.wang@siat.ac.cn (Y.W.)
† These authors contributed equally to this work.

Table S1. The molecule library of traditional Chinese formulas and Chinese patent drugs

Chinese formulas and Chinese patent drugs	Herbs (Latin name/ Herbal plant name if possible)	Herbs (Chinese Pin Yin)	main chemical components
Angong niuhuang pill	borneolum syntheticum	bingpian	alphitolic acid, camphor, caryophyllene, d-borneol, dipterocarpol, dryobalanone, erythrodiol, humulene, isoborneol, oleanolic acid
	bos taurus domesticus gmel	niuhuang	alanine, bilirubin, carotene, chenodeoxycholic acid, cholesterin, cholic acid, deoxycholic acid, ergosterol, glycine, lecithin, taurine
	bubali cornu	shuiniujiao	alanine, arginine, aspartic acid, glutamic acid, glycine, histidine, isoleucine, l-cystine, leucine, lysine, methionine, phenylalanine, proline, serine, threonine, tyrosine
	coptidis rhizoma/*Coptis chinensis* Franch.	huanglian	(+)-5,5'-dimethoxylariciresinol, berberine, columbamine, coptisine, epiberberine, jatrorrhizine, lanicepside b, palmatine, pinoresinol, secoisolariciresinol
	curcumae radix/*Curcuma aromatica* Salisb.	yujin	6-methyl-7-(3-oxobutyl)-bicyclo[4.1.0]heptan-3-one, curcumenol, curcumin, curcumin iii, demethoxy-curcumin, ethyl ferulate, ferulic acid, isocurcumenol
	gardeniae fructus/*Gardenia jasminoides* J.Ellis	zhizi	6-o-b-d-glucopyranosyl-d-glucose, beta-d-gentiobiosyl crocetin, crocetin gentiobiosylglucosyl ester, crocin 4, gardenoside, genipin 1-gentiobioside, geniposide, geniposidic acid, shanzhiside
	margarita	zhenzhu	alanine, glutamic acid, glycine, leucine, methionine, taurine
	moschus	shexiang	cholest-4-en-3-one, cholesterin, diolein, muscone, muscopyridine
	scutellariae radix/*Scutellaria baicalensis* Georgi	huangqin	baicalein, beta-caryophyllene, beta-patchoulene, chrysin, isoprene, oroxindin, oroxylin a
Da yuan decoction	anemarrhenae rhizoma/*Anemarrhena asphodeloides* Bge.	zhimu	mangiferin, timosaponin b ii, timosaponin a i, timosaponin a iii
	arecae semen/*Areca catechu* L.	binglang	arecaidine, arecatannin b1, arecolidine, arecoline hydrobromide, catechin, epicatechin, galactose, guvacine, guvacoline, homoarecoline, isoguvacine, lauric acid, mannose, myristic acid, oleic acid, palmitic acid, procyanidin b1, procyanidin b2, proline, stearic acid, sucrose, tannic acid

	glycyrrhizae radix et rhizoma/*Glycyrrhiza uralensis* Fisch.	gancao	glabridin, glycyrrhizin, isoliquiritigenin, licorice saponin a3, licorice saponin c2, licorice saponin e2, licoricidin, uralsaponin b, glycyrrhizic acid
	magnoliae officinalis cortex/*Magnolia officinalis* Rehd. et Wils	houpu	(z)-1-methoxy-3,7-dimethylocta-2,6-diene, caryophyllene, cineole, honokiol, magnolol
	paeoniae radix alba/*Paeonia lactiflora* Pall.	baishao	2'-o-benzoylpaeoniflorin, ac1l4r53, albiflorin, benzoylpaeoniflorin, dibutyl phthalate, ecliptasaponin a, ethyl gallate, kaempferol, loganin, paeoniflorin, saikosaponin b2, thymol-beta-d-glucoside
	scutellariae radix/Scutellaria baicalensis Georgi	huangqin	baicalein, beta-caryophyllene, beta-patchoulene, chrysin, isoprene, oroxindin, oroxylin a
	tsaoko fructus/*Amomum tsao-ko* Crevost et Lemaire	caoguo	2-undecenal, alpha-pinene, alpha-terpineol, beta-pinene, camphor, cineole, citral, decanal, geraniol, linalool, nerolidol, sabinene
Huanglian jiedu decoction	coptidis rhizoma/*Coptis chinensis* Franch.	huanglian	(+)-5,5'-dimethoxylariciresinol, berberine, columbamine, coptisine, epiberberine, jatrorrhizine, lanicepside b, palmatine, pinoresinol, secoisolariciresinol
	gardeniae fructus/*Gardenia jasminoides* J.Ellis	zhizi	6-o-b-d-glucopyranosyl-d-glucose, beta-d-gentiobiosyl crocetin, crocetin gentiobiosylglucosyl ester, crocin 4, gardenoside, genipin 1-gentiobioside, geniposide, geniposidic acid, shanzhiside
	Phellodendri Chinensis Cortex/Phellodendron chinense Schneid.	huangbai	berberine, candicine, hyperoside, jatrorrhizine, limonin, obacunone, palmatine, phellodendrine
	scutellariae radix/S*cutellaria baicalensis* Georgi	huangqin	baicalein, beta-caryophyllene, beta-patchoulene, chrysin, isoprene, oroxindin, oroxylin a
Jiedu huoxue decoction	angelicae sinensis radix/*Angelica sinensis* (Oliv.) Diels	danggui	4-acetamino phenol, anisic acid, beta-phellandrene, butylidene phthalide, ferulic acid, isoeugenol, niacin, o-cresol, vanillin
	Aurantia fructus/ *Citrus aurantium* L.	zhike	alpha-pinene, beta-pinene, caryophyllene, limonene, linalool, myrcene
	bupleuri radix/*Bupleurum chinense* DC.	chaihu	20-hexadecanoylingenol, 6-o-vanilloylajugol, albiflorin, capsaicin, gallic acid, glycyrrhizic acid, hesperidin, luteolin, naringin, nvr10-001e2, quercetin, quercetin chloride, saikosaponin a, saikosaponin b1, saikosaponin d, saikosaponin k, saikosaponin v, thymonin

	carthami flos/ *Carthamus tinctorius* L.	honghua	carthamin, catechol, chlorogenic acid, dopa, safflomin a
	forsythiae fructus/Forsythia suspensa (Thunb.) Vahl	lianqiao	alpha-pinene, beta-ocimene, beta-phellandrene, beta-pinene, betulinic-acid, borneol, camphene, camphor, gamma-terpinene, geranial, matairesinoside, myrcene, oleanolicacid, p-cymene, phillygenin, phillyrin, pinoresinol, ursolic acid
	glycyrrhizae radix et rhizoma/Glycyrrhiza uralensis Fisch.	gancao	glabridin, glycyrrhizin, isoliquiritigenin, licorice saponin a3, licorice saponin c2, licorice saponin e2, licoricidin, uralsaponin b, glycyrrhizic acid
	persicae semen/ *Prunus persica* (L.) Batsch	taoren	amygdalin, campesterol, chlorogenic acid, glucose, prunasin, sitosterol
	puerariae lobatae radix/ *Pueraria lobata* (Willd.) Ohwi	gegen	beta-d-glucose, daidzein, docosanoic acid, lupenone, puerarin, sitosterol, soyasaponin i
	radix padoniae rubra/ *Paeonia lactiflora* Pall.	chishao	albiflorin, benzoyl oxypaeoniflorin, benzoylpaeoniflorin, betulinic-acid, catechin, d-catechin, epifriedelanol, friedelin, gallic acid, gallotannin 23, hederagenin, kaempferol, lactinolide, oleanolicacid, oxypaeoniflorin, paeoniflorigenone, paeoniflorin, paeonilactone a, paeonilactone b, paeonilactone c, palbinone
	Rehmanniae Radix/ *Rehmannia glutinosa* Libosch.	shengdihuang	beta-sitosterol, campesterol, catalpol, mannitol, rehmannin
Ma xing shi gan deccoction	ephedrae herba/*Ephedra sinica* Stapf	mahuang	d-pseudoephedrine, l-ephedrine, l-methylephedrine, norephedrine, norpseudoephedrine
	glycyrrhizae radix et rhizoma/*Glycyrrhiza uralensis* Fisch.	gancao	glabridin, glycyrrhizin, isoliquiritigenin, licorice saponin a3, licorice saponin c2, licorice saponin e2, licoricidin, uralsaponin b, glycyrrhizic acid
	semen armeniacae amarum/*Prunus armeniaca L. var. ansu* Masim.	kuxingren	amygdalin, prunasin
Ma xing yi gan deccoction	coicis semen/ *Coix lacryma-jobi L. var. ma-yuen* (Roman.) Stapf	yiyiren	coixenolide, coixol, linolein, myristic acid, oleic acid, palmitic acid, palmitin, stearate, stearic acid, stigmasterol, triolein
	ephedrae herba/*Ephedra sinica* Stapf	mahuang	d-pseudoephedrine<br>l-ephedrine<br>l-methylephedrine<br>norephedrine<br>norpseudoephedrine
	glycyrrhizae radix et rhizoma/*Glycyrrhiza uralensis* Fisch.	gancao	glabridin, glycyrrhizin, isoliquiritigenin, licorice saponin a3, licorice saponin c2, licorice saponin e2, licoricidin, uralsaponin b, glycyrrhizic acid

	semen armeniacae amarum/*Prunus armeniaca* L. var. ansu Masim.	kuxingren	amygdalin, prunasin
Shengjiang powder	bombyx batryticatus	baijiangcan	3-hydroxykynurenine, ammonium oxalate, aspartic acid, bassianin, beauverolide d, beauverolide ea, beauverolide f, beauverolide h, beauverolide ja, ecdysterone, leucine, lysine, oxalic acid, palmitic acid
	cicadaeperiostracum	chantui	acetylglucosamine, alanine, aspartic acid, erythropterin, glutamic acid, isoxanthopterin, l-valine, proline
	curcumae longae rhizome/ *Curcuma longa* L.	jianghuang	alpha-curcumene, alpha-pinene, alpha-terpinene, ar-curcumene, ar-turmerone, beta-curcumene, beta-pinene, beta-turmerone, bisdemethoxycurcumin, borneol, caryophyllene, cineole, curcumin, curcumol, curdione, curzerenone, demethoxy-curcumin, dihydrocurcumin, gamma-curcumene, germacrone, limonene, linalool, turmeronol
	rhei radix et rhizoma/*Rheum palmatum* L.	dahuang	c, sennoside a, sennoside b, sennoside c monoglucoside, rheidin a, rheidin b, rheidin c, rhein, rheinoside a, rheinoside aloe-emodin, beta-sitosterol, chrysophanol, cinchophen, emodin, emodin-8-glucoside, eupatin, palmidin a, palmidin b, palmidin c, physcion, physcion
Sini jia renshen deccoction	aconiti lateralis radix praepar/ *Aconitum carmichaelii* Debx.	fuzi	aconine, benzoylaconine, benzoylmesaconine, hypaconine, mesaconine
	ginseng radix et rhizome/ *Panax ginseng* C. A. Mey.	renshen	dammar-24-ene-3, notoginsenoside r4, protopanaxatriol
	glycyrrhizae radix et rhizoma/*Glycyrrhiza uralensis* Fisch.	gancao	glabridin, glycyrrhizin, isoliquiritigenin, licorice saponin a3, licorice saponin c2, licorice saponin e2, licoricidin, uralsaponin b, glycyrrhizic acid
	zingiberis rhizome/ *Zingiber officinale* (Willd.) Rosc.	ganjiang	10-gingerol, 6-dehydrogingerdione, 6-gingerol, 6-shogaol, beta-sitosteryl palmitate, dibutyl phthalate, hexahydrocurcumin, paradol, tetracosanoic acid, triacontanoic acid, zingerone
Suhexiang pill	aquilariae lignum resinat/*Aquilaria sinensis* (Lour.) Gilg	chenxiang	agarospirol, baimuxinal, baimuxinic acid, baimuxinol, caryophyllene oxide
	atractylodes macrocephala koidz/ *Atractylodes macrocephala* Koidz.	baizhu	beta-elemene
	aucklandiae radix/*Aucklandia lappa* Decne.	muxiang	bicyclo[2.2.1]hept-2-ene, borneol, decane, octane, phenylethanol

	benzoinum	anxixiang	benzaldehyde, benzyl cinnamate, cinnamaldehyde, cinnamyl cinnamate, styrene
	borneolum syntheticum	bingpian	alphitolic acid, beta-elemene, borneol, camphor, caryophyllene, d-borneol, dipterocarpol, dryobalanone, erythrodiol, humulene, isoborneol, oleanolic acid
	bubali cornu	shuiniujiao	alanine, arginine, aspartic acid, glutamic acid, glycine, histidine, isoleucine, l-cystine, leucine, lysine, methionine, phenylalanine, proline, serine, threonine, tyrosine
	caryophylli flos/*Eugenia caryophyllata* Thunb.	dingxiang	benzaldehyde, benzyl acetate, beta-caryophyllene, eugenol, eugenol acetate
	chebulae fructus	hezirou	beta-sitosterol, chebulagic acid, chebulinic acid, corilagin, gallic acid, pentagalloylglucose, quinic acid, sennoside a, shikimic acid
	cyperi rhizome/ *Cyperus rotundus* L.	xiangfu	alpha-cyperone, beta-selinene, cyperene, cyperol, terpinen-4-ol
	moschus	shexiang	cholest-4-en-3-one, cholesterin, diolein, muscone, muscopyridine
	olibanum	ruxiang	alpha-boswellic acid, beta-boswellic acid, elemonic acid, glycerol monopalmitate, linalool
	piperis longi fructus/ *Piper longum* L.	biba	2-phenylpropionic acid, beta-sitosterol, methyl piperate, pellitorine, piperanine, piperine, pipernonaline
	santali albi lignum/*Santalum albm* L.	tanxiang	alpha-curcumene, limonene, nuciferol, santalene, santalol
	styrax	suhexiang	(1,4a-dimethyl-7-propan-2-yl-2,3,4,4b,5,6,7,8,8a,9,10,10a-dodecahydrophenanthren-1-yl)methanol, benzyl benzoate, benzyl cinnamate, caryophyllene, myrcene
Xuanbai chengqi decoction	rhei radix et rhizoma/*Rheum palmatum* L.	dahuang	c, sennoside a, sennoside b, sennoside c monoglucoside, rheidin a, rheidin b, rheidin c, rhein, rheinoside a, rheinoside aloe-emodin, beta-sitosterol, chrysophanol, cinchophen, emodin, emodin-8-glucoside, eupatin, palmidin a, palmidin b, palmidin c, physcion, physcion
	semen armeniacae amarum/*Prunus armeniaca* L. var. ansu Masim.	kuxingren	amygdalin, prunasin
	trichosanthis fructus	gualou	alanine, arginine, campesterol, cerotic acid, glycine, isoleucine, leucine, l-valine, lysine, oleic acid, palmitic acid, punicic acid, tetracosanoic acid
Yin qiao powder	arctii fructus/*Arctium lappa* L.	niubangzi	arachidic acid, arctigenin, arctiin, glucose, lappaol b, matairesinol,

			menthone, oleic acid, palmitic acid, stearic acid, trachelogenin
	bambusae caulis in taenias/ *Bambusa tuldoides* Munro	zhuye	d-fucose, isoorientin, kaempferol-3-o-beta-d-glucoside, kaempferol-3-rutinoside, luteolin-7-o-glucoside, lysine, mannose, orientin, rhamnose, rutin, trans-2-hexenal, vitexin
	forsythiae fructus/*Forsythia suspensa* (Thunb.) Vahl	lianqiao	alpha-pinene, beta-ocimene, beta-phellandrene, beta-pinene, betulinic-acid, borneol, camphene, camphor, gamma-terpinene, geranial, matairesinoside, myrcene, oleanolicacid, p-cymene, phillygenin, phillyrin, pinoresinol, ursolic acid
	glycyrrhizae radix et rhizoma/*Glycyrrhiza uralensis* Fisch.	gancao	glabridin, glycyrrhizin, isoliquiritigenin, licorice saponin a3, licorice saponin c2, licorice saponin e2, licoricidin, uralsaponin b, glycyrrhizic acid
	lonicerae japonicae/ *Lonicera japonica* Thunb.	jinyinhua	beta-sitosterol, chlorogenic acid, isochlorogenic acid, linalool, stigmasterol, trans-trans-farnesol
	menthae haplocalycis herba/ *Mentha haplocalyx* Briq.	bohe	carvone, linalool, l-menthol, piperitone, p-menthan-3-one
	platycodonis radix/ *Platycodon grandiflorum* (Jacq.) A. DC.	jiegeng	1-undecene, isopropyl formate, linolenic acid, luteolin-7-o-rutinoside, oleic acid, palmitic acid, platycodin a, platycodin d2, quercetin-7-o-rutinoside, quercimeritrin, stearic acid, taxifolin
	schizonepetae herba/ *Schizonepeta tenuifolia* Briq.	jingjiesui	apigenin, beta-sitosterol, hesperidin, luteolin, p-coumaric-acid, tilianin, ursolic acid
	sojae semen praeparatum/ *Glycine max* (L.) Merr.	dandouchi	asparagine, carotene, choline, daidzein, daidzin, genistin, glycine, niacin, syringic acid, xanthine
Zi xue powder	aquilariae lignum resinat/*Aquilaria sinensis* (Lour.) Gilg	chenxiang	agarospirol, baimuxinal, baimuxinic acid, baimuxinol, caryophyllene oxide
	aucklandiae radix/*Aucklandia lappa* Decne.	muxiang	bicyclo[2.2.1]hept-2-ene, borneol, decane, octane, phenylethanol
	bubali cornu	shuiniujiao	alanine, arginine, aspartic acid, glutamic acid, glycine, histidine, isoleucine, l-cystine, leucine, lysine, methionine, phenylalanine, proline, serine, threonine, tyrosine
	caryophylli flos/*Eugenia caryophyllata* Thunb.	dingxiang	benzaldehyde, benzyl acetate, beta-caryophyllene, eugenol, eugenol acetate
	cimicifugae rhizoma	shengma	3-methyl-5 (2,6, 6-trimethyl-1-cyclohexene) -2-pentenoic acid, 9,17-octadecadienal, beta-elemene, linoleyl alcohol, palmitic acid
	glycyrrhizae radix et rhizoma/Glycyrrhiza uralensis Fisch.	gancao	glabridin, glycyrrhizin, isoliquiritigenin, licorice saponin a3, licorice saponin c2,

			licorice saponin e2, licoricidin, uralsaponin b, glycyrrhizic acid
	moschus	shexiang	cholest-4-en-3-one, cholesterin, diolein, muscone, muscopyridine
	saiga tatarica linnaeus	lingyangjiao	aspartic acid, dipalmitoyl cephalin, glutamic acid, lecithins, leucine, lysine, phenylalanine, phosphatidylserine, serine, tyrosine
	scrophulariae radix/ *Scrophularia ningpoensis* Hensl.	xuanshen	acteoside, angoroside c, cistanoside d, cistanoside f, ningposide a, ningposide b, ningposide c, ningposide d, sibirioside a

Table S2. Target protein setup

Target protein	Protein ID in PDB	grid box radius
ACE2	1R4L	25Å
SARS-CoV-2 chimeric RBD	6VW1	25Å
RdRp	6M71	25Å
Mpro	6LU7	25Å

Table S3. GO enrichment analysis

Term	Count	%	PValue
GO:0006954~inflammatory response	5	7.04225	0.00235
GO:0006955~immune response	4	5.63380	0.00966
GO:0007009~plasma membrane organization	2	2.81690	0.04729
GO:0035329~hippo signaling	2	2.81690	0.04729
GO:0001974~blood vessel remodeling	2	2.81690	0.08355
GO:0031623~receptor internalization	2	2.81690	0.08946
GO:0008543~fibroblast growth factor receptor signaling pathway	2	2.81690	0.09240
GO:0010629~negative regulation of gene expression	3	4.22535	0.09379
